# Managing an infected femoral artery pseudoaneurysm after thoraco-bifemoral bypass with an innovative configuration

**DOI:** 10.1016/j.jvscit.2022.08.023

**Published:** 2022-09-08

**Authors:** Mu’ath Adlouni, Ezra Y. Koh, Maham Rahimi

**Affiliations:** a College of Medicine, Texas A&M University, Bryan, TX; b College of Engineering, Texas A&M University, Bryan, TX; c Department of Cardiovascular Surgery, Houston Methodist Hospital, Houston, TX

**Keywords:** Complications, Femoral artery, Infected, Pelvis, Pseudoaneurysm, Vessel

## Abstract

An infected femoral artery pseudoaneurysm after aortic reconstruction is a devastating surgical complication associated with the morbidity of limb loss and pelvic ischemia with a reinfection rate of ≤10%. In the present case report, we have described a unique approach for an infected femoral pseudoaneurysm after thoraco-bifemoral bypass using an innovative configuration, in addition to an obturator bypass technique, in a patient with a complex vascular history. This unique approach made use of an existing limb of a thoraco-bifemoral bypass graft to provide inflow to two outflow conduits, the external iliac artery and superficial femoral artery, allowing for preservation of both pelvic and lower extremity perfusion.

Anastomotic pseudoaneurysms (PSAs) are devastating complications of vascular reconstruction procedures that often occur in the femoral region, with an incidence of 2% to 5%.^1^^,^^2^ Management of this complication requires an early diagnosis and surgical reconstruction because emergent arterial reconstruction has been associated with significantly worse outcomes.^3^^,^^4^ Moreover, management of femoral artery anastomotic PSAs requires additional attention owing to the potential for pelvic ischemia, especially after extra-anatomic bypass. We present a case of a successfully treated infected anastomotic femoral artery PSA with an extra-anatomic obturator bypass, maintaining the pelvic and lower extremity perfusion using a unique configuration. The patient provided written informed consent for the report of her case details and imaging studies.

## Case report

**Fig 1:**
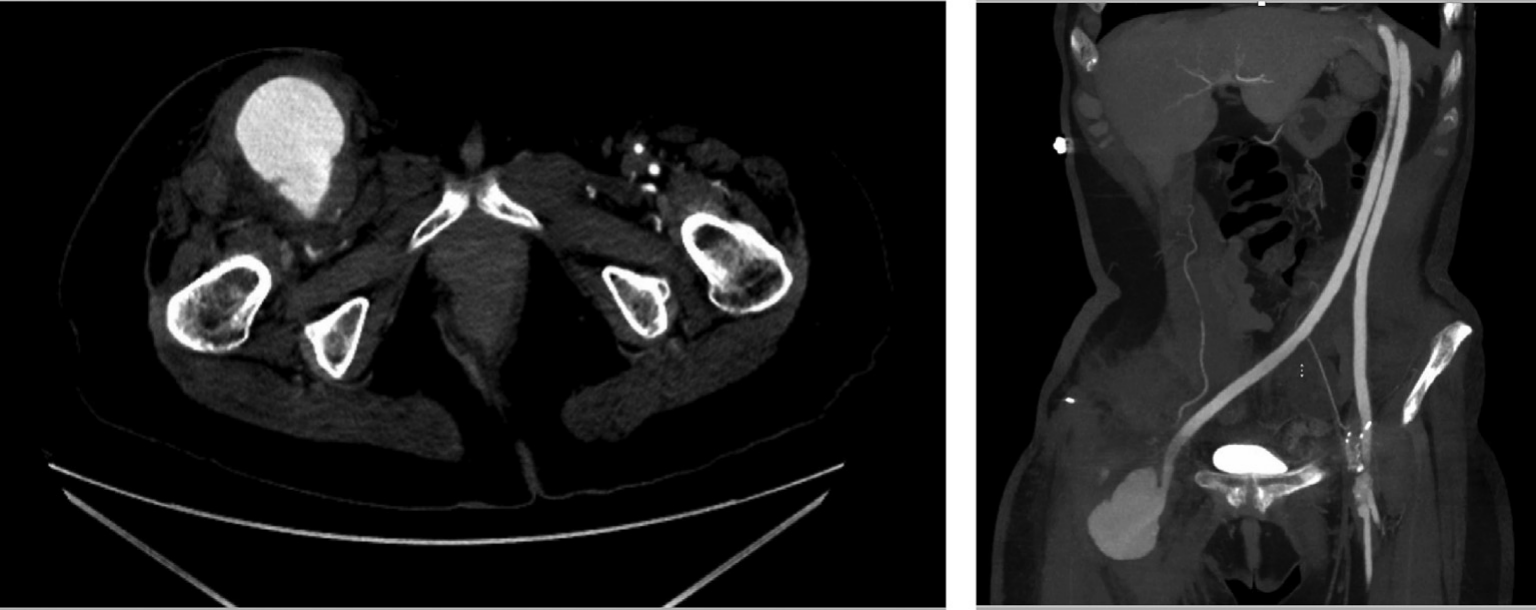
Computed tomography images of right femoral artery pseudoaneurysm (PSA).

A 60-year-old woman with a medical history significant for peripheral vascular disease with multiple revascularization attempts, hypertension, hyperlipidemia, chronic kidney disease, and previous tobacco abuse had presented with a right femoral anastomotic pseudoaneurysm after a recent thoraco-bifemoral bypass. She had a complex surgical history with multiple attempts at aortoiliac revascularization at an outside institution that included a thrombosed femorofemoral bypass and aortobifemoral bypass due to poor inflow. She was referred to our institution for treatment of lower limb ischemia with rest pain. She subsequently underwent thoraco-bifemoral bypass and was discharged without any complications. She returned 3 months postoperatively with right groin pain, a pulsatile mass, and erythema with only dorsalis pedis and posterior tibial artery Doppler signals in the right lower extremity. Computed tomography angiography (CTA) revealed a patent thoraco-bifemoral bypass with a 7 × 6 × 4-cm PSA at the right common femoral anastomosis with no evidence of fluid extension around the limb of the bypass proximally, a patent right superficial femoral artery (SFA), and an occluded left internal iliac artery (IIA; Fig 1).

## Surgical technique

The patient was taken to the operating room for urgent revascularization and was prepared from the chin down, including the circumferential bilateral lower extremities. Ultrasound was used to identify the right SFA, and an incision was made over the right medial thigh. Dissection was performed until the sartorius muscle was identified. The fascia medial to the sartorius was then incised to expose the SFA and achieve proximal and distal control using vessel loops. A micropuncture kit and a 5F sheath catheter (Cook Medical, Inc, Bloomington, IN) were then used to perform a right lower extremity angiogram. This was necessary to ensure adequate perfusion to the lower extremity because CTA had failed to visualize the tibial vessels owing to the large size of the PSA diverting the contrast. The angiogram revealed adequate perfusion to the lower extremity with three-vessel runoff.

Next, we proceeded with a right lower quadrant Gibson incision, and dissection was continued toward the retroperitoneal space to expose the external iliac artery (EIA), which was controlled via vessel loops. A graft tunneling device was then used to pass a Y-shaped, 8-mm Propaten polytetrafluoroethylene (PTFE) graft (W.L. Gore & Associates, Flagstaff, AZ) from the right retroperitoneal space through the obturator foramen into the right thigh incision.

Next, the right limb of the thoraco-bifemoral bypass graft was identified inferior to the rectus abdominis muscle. A 5-cm segment of the thoraco-bifemoral bypass graft was resected and its distal end suture ligated. The femorofemoral bypass was also identified in this space and transected. The proximal end of the thoraco-bifemoral bypass graft was used as the inflow branch for the Y-shaped Propaten PTFE graft (W.L. Gore & Associates). We ensured that the right limb of the thoracofemoral graft and the branch Y-shaped Propaten PTFE graft were without kinks in the retroperitoneal space.

**Fig 2:**
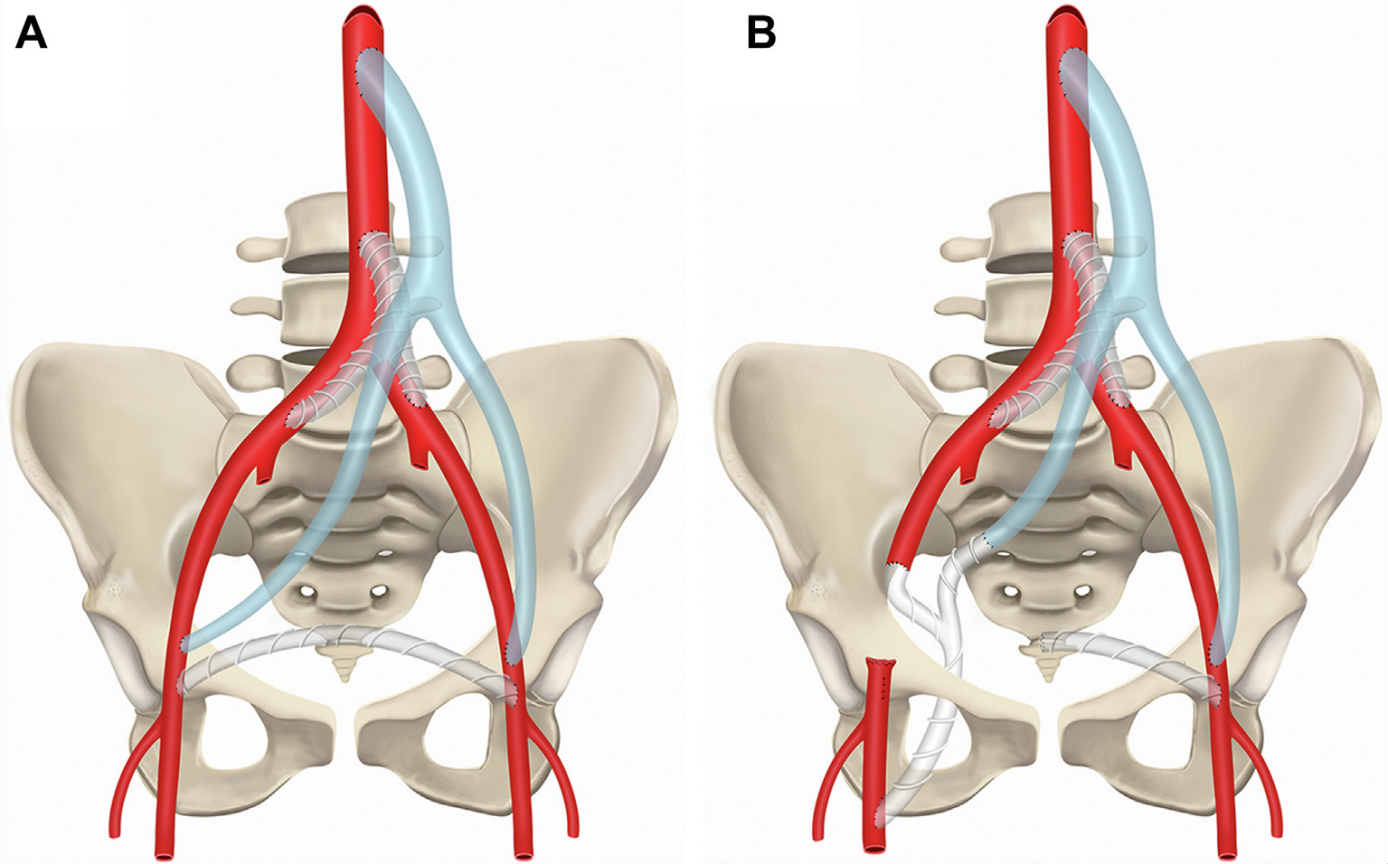
**A,** Patient’s vascular history and graft configuration, which included an aortobifemoral graft, a thoraco-bifemoral graft, and a femorofemoral graft. **B,** Configuration of the axillobifemoral polytetrafluoroethylene (PTFE) obturator graft after surgery with proximal anastomoses to the right external iliac artery (EIA) and right limb of the thoraco-bifemoral graft.

The right EIA was then clamped and transected, and the proximal end was suture ligated to isolate the PSA. The distal end of the EIA was then sutured in an end-to-end fashion using 5-0 Prolene suture to the corresponding branch of Y-shaped PTFE graft. Next, the tunneled limb of the Y-shaped Propaten PTFE graft (W.L. Gore & Associates) was anastomosed to the right SFA. The SFA arteriotomy was created, and an end-to-side anastomosis was performed using 5-0 Prolene suture. The patient's vascular history and the graft configuration, consisting of an aortobifemoral graft, a thoraco-bifemoral graft, and a femorofemoral graft, are shown in Fig 2, *A*. The configuration of the axillobifemoral PTFE graft after surgery, with one limb through the obturator foramen, the second limb anastomosed to the EIA, and third limb coming anteriorly to form the anastomosis to the right limb of the thoraco-bifemoral graft, is shown in Fig 2, *B*.

After closing the incisions, we focused on the groin. The PSA was no longer pulsatile and was excised from the groin, as was the blood redirected through the bypass. We then washed the cavity with rifampin solution, and the profunda femoris artery was suture ligated with 3-0 Prolene. Finally, antibiotic beads were placed, and the skin was closed with running 3-0 nylon sutures.

## Postoperative course

The patient returned to the operating room on postoperative day 4 for retrieval of the right groin antibiotic beads, followed by wound vacuum placement. The results from blood cultures, fungal cultures, and gram stains were all negative, with only the tissue cultures returning positive for diphtheroid bacilli. The patient was discharged 1 week later after a total postoperative hospital stay of 14 days. She received intravenous vancomycin and piperacillin-tazobactam for 6 weeks, followed by lifelong oral amoxicillin-clavulanate. She followed up at our office at 2 weeks, 1 month, and 6 months after surgery. She was able to ambulate with a walker, and her groin wounds had healed well. She has continued to have no symptoms of pelvic ischemia or limb claudication with a palpable pedal pulse. Because of her chronic kidney disease, CTA was not obtained, and her noninvasive vascular studies indicated no evidence of inflow, conduit, or outflow stenosis.

## Discussion

Surgical treatment of infected femoral PSAs involves revascularization in situ using conduits such as the femoral vein, rifampin-soaked Dacron, or CryoVein (CyroLife, Kennesaw, GA) or extra-anatomic bypass via a route remote from the infected field and ligation of the common femoral artery.^5^, ^6^, ^7^ The extra-anatomic bypass involves tunneling an artificial PTFE graft through the obturator foramen and using the EIA as the inflow and the distal SFA as the outflow. This common approach for achieving reperfusion of the distal lower extremity is more advantageous, because Bath et al^8^ demonstrated promising limb salvage and patency rates in their study of the long-term outcomes of obturator canal bypass. However, despite the effectiveness of revascularization with extra-anatomic bypass, it would have failed to mitigate our patient's risk of pelvic ischemia. In addition, the alternative treatment, in situ revascularization, was not a viable option owing to the risk of ongoing infection in the right groin disrupting the anastomosis again.

Pelvic ischemia is a devastating complication after aortoiliac revascularization. Of the most reported complications after endovascular intervention, pelvic ischemia has had a reported incidence of 28% for patients after unilateral IIA interruption and 42% after bilateral IIA interruption.^9^ To the best of our knowledge, techniques to preserve pelvic perfusion after managing an infected femoral PSA occurring after thoraco-bifemoral bypass have not been previously described. Therefore, consideration of pelvic ischemia was of the utmost importance owing to the significant effect that symptoms such as sciatic nerve palsy, gluteal necrosis, and buttock claudication will have on patient quality of life, as described by Jean-Baptiste et al.^10^

In the present report, we have described a novel technique for the management of an infected femoral artery anastomosis after thoraco-bifemoral bypass to allow for perfusion of both the pelvis and the distal extremity via two outlet conduits, the EIA and SFA, providing retrograde perfusion to the pelvis and antegrade perfusion to the lower extremity. Maintenance of the retrograde EIA flow into the right IIA was crucial for the prevention of pelvic ischemia in the setting of a chronically occluded left IIA. In addition, perfusion of the distal extremity through the SFA ensured adequate blood flow to the right lower extremity.

## Conclusions

Pelvic ischemia after aortoiliac reconstruction is a devastating complication associated with high morbidity and mortality. It is crucial to the preserve pelvic perfusion whenever possible. Using the approach we have described in the present report, we were able to successfully repair an infected anastomotic femoral artery PSA with partial vascular graft excision and maintain pelvic and lower extremity perfusion.
